# Evaluating the Efficacies of Carbapenem/β-Lactamase Inhibitors Against Carbapenem-Resistant Gram-Negative Bacteria *in vitro* and *in vivo*

**DOI:** 10.3389/fmicb.2019.00933

**Published:** 2019-04-30

**Authors:** Bassam El Hafi, Sari S. Rasheed, Antoine G. Abou Fayad, George F. Araj, Ghassan M. Matar

**Affiliations:** ^1^Department of Experimental Pathology, Immunology and Microbiology, American University of Beirut, Beirut, Lebanon; ^2^Center for Infectious Diseases Research, American University of Beirut, Beirut, Lebanon; ^3^Department of Pathology and Laboratory Medicine, American University of Beirut Medical Center, Beirut, Lebanon

**Keywords:** OXA-48, NDM-1, KPC, carbapenem, Avibactam, Relebactam, calcium-EDTA, antimicrobial resistance

## Abstract

**Background:**

Carbapenem-resistant Gram-negative bacteria are a major clinical concern as they cause virtually untreatable infections since carbapenems are among the last-resort antimicrobial agents. β-Lactamases implicated in carbapenem resistance include KPC, NDM, and OXA-type carbapenemases. Antimicrobial combination therapy is the current treatment approach against carbapenem resistance in order to limit the excessive use of colistin; however, its advantages over monotherapy remain debatable. An alternative treatment strategy would be the use of carbapenem/β-lactamase inhibitor (βLI) combinations. In this study, we assessed the *in vitro* and *in vivo* phenotypic and molecular efficacies of three βLIs when combined with different carbapenems against carbapenem-resistant Gram-negative clinical isolates. The chosen βLIs were (1) Avibactam, against OXA-type carbapenemases, (2) calcium-EDTA, against NDM-1, and (3) Relebactam, against KPC-2.

**Methods:**

Six *Acinetobacter baumannii* clinical isolates were screened for *bla*_OXA-23-like_, *bla*_OXA-24/40_, *bla*_OXA-51-like_, *bla*_OXA-58_, and *bla*_OXA-143-like_, and eight *Enterobacteriaceae* clinical isolates were screened for *bla*_OXA-48_, *bla*_NDM-1_, and *bla*_KPC-2_. The minimal inhibitory concentrations of Imipenem (IPM), Ertapenem (ETP), and Meropenem (MEM) with corresponding βLIs for each isolate were determined. The efficacy of the most suitable *in vitro* treatment option against each of *bla*_OXA-48_, *bla*_NDM-1_, and *bla*_KPC-2_ was assessed via survival studies in a BALB/c murine infection model. Finally, RT-qPCR was performed to assess the molecular response of the genes of resistance to the carbapenem/βLI combinations used under both *in vitro* and *in vivo* settings.

**Results:**

Combining MEM, IPM, and ETP with the corresponding βLIs restored the isolates’ susceptibilities to those antimicrobial agents in 66.7%, 57.1%, and 30.8% of the samples, respectively. Survival studies in mice revealed 100% survival rates when MEM was combined with either Avibactam or Relebactam against *bla*_OXA-48_ and *bla*_KPC-2_, respectively. RT-qPCR demonstrated the consistent overexpression of *bla*_OXA-48_ upon treatment, without hindering Avibactam’s activity, while *bla*_NDM-1_ and *bla*_KPC-2_ experienced variable expression levels upon treatment under *in vitro* and *in vivo* settings despite their effective phenotypic results.

**Conclusion:**

New carbapenem/βLI combinations may be viable alternatives to antimicrobial combination therapy as they displayed high efficacy *in vitro* and *in vivo.* Meropenem/Avibactam and Meropenem/Relebactam should be tested on larger sample sizes with different carbapenemases before progressing further in its preclinical development.

## Introduction

Carbapenem resistant Gram-negative bacteria have been gradually increasing in prevalence in recent years. In the United States, the latest CDC Antibiotic Resistance Threat Report indicates that Carbapenem-Resistant *Enterobacteriaceae* (CREs) are responsible for 9,000 annual nosocomial infections, with a 6.67% mortality rate; a potentially underestimated percentage due to different definitions of CRE infections (Livorsi et al., 2018). The same report also estimates 7,300 annual multidrug-resistant (MDR) *Acinetobacter baumannii* infections; with a 6.85% mortality rate. In Lebanon, the most recent nation-wide survey indicates that around 2% of *Enterobacteriaceae* isolates identified over the past few years were Imipenem-resistant, while that percentage was much higher among *Acinetobacter* spp. at 82.4% (Chamoun et al., 2016). At the American University of Beirut Medical Center (AUBMC), the prevalence of CREs has doubled since 2015, reaching 11%, while carbapenem resistance among *A. baumannii* isolates has remained high beyond 75% during the same time period (Araj and Zaatari, 2015, 2018).

Carbapenem resistance can manifest through several mechanisms. Notably, the combined effect of extended-spectrum β-lactamases (ESBLs) or AmpC-type enzymes production, coupled with increased efflux pump activity and porin loss (Baroud et al., 2013). However, the main mechanism of resistance to carbapenems is through the expression of chromosomal or plasmid-mediated carbapenem-hydrolyzing β-lactamases such as *Klebsiella pneumoniae* carbapenemases (KPC), OXA-type carbapenemases, and New Delhi metallo-β-lactamases (Lapuebla et al., 2015) (Meletis, 2016). KPC and OXA-type carbapenemases are families of Ambler Class A and Class D serine β-lactamases, respectively, that contain a serine moiety in their active sites (Sahuquillo-Arce et al., 2015). Among the KPC family, KPC-2 and KPC-3 are the most commonly encountered between the 20-plus variant KPCs (Djahmi et al., 2014; Sahuquillo-Arce et al., 2015; Satlin et al., 2017). The OXA-type carbapenemases are grouped into nine clusters with 1, 2, 3, and 4 being associated with *A. baumannii*, and include the subfamilies OXA-23, OXA-51, OXA-24/40, and OXA-58, while cluster 6, being associated with *Enterobacteriaceae*, comprises the subfamily OXA-48 (Woodford et al., 2006; Queenan and Bush, 2007). On the other hand, NDM is a family of Ambler Class B metallo-β-lactamases that contain a divalent cation in their active site (Sahuquillo-Arce et al., 2015) with NDM-1 being the most prominent member (Nordmann et al., 2011).

Treating carbapenem-resistant Gram-negative bacteria poses a major clinical challenge as carbapenems are among the last-resort antimicrobial agents to be used, and CREs along with MDR-*A. baumannii* can cause terminal infections ranging from upper and lower respiratory, wound, bloodstream and cerebrospinal fluid infections in the case of *A. baumannii* (Queenan et al., 2012), to complicated intra-abdominal infections, sepsis, and meningitis, in the case of CREs (Murray et al., 2016; Yu and Chuang, 2016). The current recommendation to treat carbapenem-resistant Gram-negative infections involves the use of antimicrobial combination therapy (The Medical Letter, 2013). This approach is mostly guided by the lack of new classes of antimicrobial agents that can overcome such resistance since it is usually compounded with fluoroquinolone as well as aminoglycoside resistances within the same isolate (Meletis, 2016). Consequently, nephrotoxic antimicrobial agents such as polymyxins have to be combined with tetracyclines, such as tigecycline (Meletis, 2016). However, the efficacy of antimicrobial combination therapy in comparison to monotherapy has been a topic of debate in the literature. One study concluded that combination therapy improved the survival rates of bloodstream infection patients and decreased their mortality rates by 20.2% (*p* = 0.02) when compared to monotherapy (Tumbarello et al., 2012). Another study conducted on 205 patients infected with KPC-producing *K. pneumoniae* determined that combination therapy decreased patient mortality rate from 40 to 19.4% when a carbapenem is used in addition to other antimicrobials (Daikos et al., 2014). However, there exists sources of bias in combination therapy reports since a lot of studies include both carbapenem-resistant and carbapenem-susceptible isolates, not to mention that they disregard empirical treatment that the patient might have taken prior to being enrolled in the study (Paul et al., 2014). Additionally, certain studies report that the use of carbapenems as part of a double or triple therapy is recommended when the MIC needed against the isolate is ≤8 μg/ml (Tzouvelekis et al., 2012; Daikos et al., 2014) whereas other studies assign that breakpoint at ≤4 μg/mL (Miyakis et al., 2011; Tängdén, 2014). Finally, combination therapy increases the cost of treatment (Kmeid et al., 2013) and exposes the bacteria to several antimicrobials that it might develop resistance to. As such, the utility of combination therapy remains off-label and largely biased. Therefore, an alternative treatment approach to carbapenem-resistant bacterial infections could be the genetically guided use of β-lactam/β-lactamase inhibitors (βLIs), namely, carbapenems/βLIs combinations.

Three βLIs were selectively chosen to target specific mechanisms of carbapenem resistance: Avibactam against OXA-type carbapenemases, Relebactam against KPC, and calcium-EDTA against NDM. First, Avibactam is a non-β-lactam-based βLI that reversibly inactivates serine carbapenemases through the covalent acylation of the β-lactamase followed by a slow deacylation step that restores the inhibitor’s core chemical structure (Ehmann et al., 2012). Avibactam is United States FDA-approved in combination with Ceftazidime (U.S. Food and Drug Administration [FDA], 2015) and is marketed as a treatment option against hospital-acquired and ventilator-associated bacterial pneumonias, and complicated intra-abdominal and urinary tract infections (Allergan, 2018). Secondly, Relebactam is also a non-β-lactam-based βLI that targets serine carbapenemases (Hirsch et al., 2012); however, it is combined with Imipenem/Cilastatin to target Imipenem-resistant bacteria (Livermore et al., 2013; Lapuebla et al., 2015; Lob et al., 2017; Karlowsky et al., 2018). Finally, calcium disodium EDTA is a divalent metal-chelating agent that is United States FDA-approved to treat acute and chronic lead poisoning (U.S. Food and Drug Administration [FDA], 2009); however, it has been described in the literature to have the capacity to chelate the divalent cations found in the active sites of metallo-β-lactamases and has shown *in vivo* efficacy against *Pseudomonas aeruginosa* and *Escherichia coli* when combined with β-lactams (Aoki et al., 2010; Yoshizumi et al., 2013).

In this study, we first aim to assess the *in vitro* and *in vivo* efficacies of carbapenems in combination with the βLIs Avibactam, Relebactam, and calcium-EDTA when targeting OXA-type carbapenemases, KPC-2, and NDM-1, respectively, and then investigate the molecular response of those genes of resistance against the carbapenem/βLI combinations.

## Materials and Methods

### Isolate Collection

The Department of Pathology and Laboratory Medicine at the American University of Beirut Medical Center (AUBMC), Beirut, Lebanon provided all of the clinical bacterial isolates included in this study with the exception of one *Salmonella* spp. isolate that was provided by the Centers for Disease Control and Prevention (CDC), Atlanta, GA, United States. A total of 14 isolates were used, including: six *A. baumannii*, five *K. pneumoniae*, two *E. coli*, and one *Salmonella* spp. isolates. In addition to those isolates, three samples of presumptive carbapenem-resistant *Pseudomonas aeruginosa* were provided. All isolates were collected as part of routine medical sampling; thus, did not require Institutional Review Board (IRB) approval nor patient consent. The labeling of each isolate can be found in Table 1.

**Table 1:** Labels of each isolate included in the study along with their detected genes of carbapenem resistance.

Bacterial species	Isolate label	Genes of carbapenem resistance							
		
		*bla*_OXA-48_	*bla*_NDM-1_	*bla*_KPC-2_	*bla*_OXA-23-*like*_	*bla*_OXA-24/40_	*bla*_OXA-51-*like*_	*bla*_OXA-58_	*bla*_OXA-143-*like*_
*Escherichia coli*	IMP 53	+	-	-					
	IMP 57	+	-	-					
*Klebsiella pneumoniae*	IMP 197	+	-	-					
	IMP 215	+	-	-					
	IMP 223	+	-	-					
	IMP 216	-	+	-					
	IMP 217	-	+	-					
*Salmonella* spp.	KPC	-	-	+					
*Acinetobacter baumannii*	ACN 2090		-		+	-	+	+	-
	ACN 2209		-		+	-	+	-	-
	ACN 2273		-		+	-	+	-	-
	ACN 2285		-		+	-	+	-	+
	ACN 2493		-		+	-	+	-	-
	ACN 3630		-		+	-	+	+	+
*Pseudomonas aeruginosa*	PSA 41		-						
	PSA 44		-						
	PSA 45		-						


*+, denotes the presence of the gene, - denotes the absence of the gene.*

### Detection of Carbapenem Resistance Genes

Total genomic DNA of each of the collected isolates was extracted from an overnight culture using the QIAamp^®^ DNA Mini Kit (QIAGEN, Germany) according to manufacturer’s instructions. Polymerase chain reaction (PCR) using TopTaq^TM^ DNA Polymerase (QIAGEN, Germany) was then utilized to amplify and detect several β-lactamase-encoding genes that are implicated in carbapenem resistance. For the *Enterobacteriaceae* isolates, *bla*_OXA-48_, *bla*_NDM-1_, and *bla*_KPC-2_ genes were tested. For the *A. baumannii* isolates, *bla*_NDM-1_, *bla*_OXA-23-like_, *bla*_OXA-24/40_, *bla*_OXA-51-like_, *bla*_OXA-58_, and *bla*_OXA-143-like_ genes were tested. For the *P. aeruginosa* isolates, *bla*_NDM-1_ was tested. The list of PCR primer sequences along with their target genes amplicon sizes are available in Table 2.

**Table 2:** List of target genes along with their PCR primer sequences and amplicon sizes.

	Target gene	PCR primer sequence (5′→3′)	Amplicon (bp)	References
PCR primers	*bla*_OXA-48_	F: TTGGTGGCATCGATTATCGG	744	Salloum et al., 2015
		R: GAGCACTTCTTTTGTGATGGC		
	*bla*_NDM-1_	F: GGAAACTGGCGACCAACG	678	Salloum et al., 2015
		R: ATGCGGGCCGTATGAGTGA		
	*bla*_OXA-23-like_	F: GATCGGATTGGAGAACCAGA	501	Woodford et al., 2006
		R: ATTTCTGACCGCATTTCCAT		
	*bla*_OXA-24/40_	F: GGTTAGTTGGCCCCCTTAAA	246	Woodford et al., 2006
		R: AGTTGAGCGAAAAGGGGATT		
	*bla*_OXA-51-like_	F: TAATGCTTTGATCGGCCTTG	353	Woodford et al., 2006
		R: TGGATTGCACTTCATCTTGG		
	*bla*_OXA-58_	F: AAGTATTGGGGCTTGTGCTG	599	Woodford et al., 2006
		R: CCCCTCTGCGCTCTACATAC		
	*bla*_OXA-143-like_	F: TGGCACTTTCAGCAGTTCCT	149	Higgins et al., 2010
		R: TAATCTTGAGGGGGCCAACC		
	*bla*_KPC-2_	F: GCAGCGGCAGCAGTTTGTTGATT	184	Salloum et al., 2015
		R: GTAGACGGCCAACACAATAGGTGC		
RT-qPCR primers	*bla*_OXA-48_	F: TTCGGCCACGGAGCAAATCAG	240	Salloum et al., 2015
		R: GATGTGGGCATATCCATATTCATCGCA		
	*bla*_NDM-1_	F: TTGGCGATCTGGTTTTCC	195	Salloum et al., 2015
		R: GGTTGATCTCCTG CTTGA		
	*bla*_KPC-2_	F: GCAGCGGCAGCAGTTTGTTGATT	184	Salloum et al., 2015
		R: GTAGACGGCCAACACAATAGGTGC		
	*rpoB*	F: TCGAAACGCCTGAAGGTC	184	Salloum et al., 2015
		R: TTGGAGTTCGCCTGAGC		


### Determination of Minimal Inhibitory Concentrations

For each of the *Enterobacteriaceae* and *A. baumannii* isolates included in this study, the minimal inhibitory concentrations (MICs) of Imipenem (as Imipenem/Cilastatin, Tienam^®^, Merck & Co., Inc., Whitehouse Station, NJ, United States), Ertapenem (Invanz^®^, Merck & Co., Inc., Whitehouse Station, NJ, United States), and Meropenem (Meronem^®^, AstraZeneca, Wilmington, DE, United States) were determined via antimicrobial broth microdilution in accordance with the Clinical and Laboratory Standards Institute (CLSI) guidelines (Clinical and Laboratory Standards Institute [CLSI], 2012). *Escherichia coli* (ATCC^®^ 25922^TM^) was used as a quality control strain (Clinical and Laboratory Standards Institute [CLSI], 2018c).

### Assessment of the *in vitro* Efficacy of the Carbapenem/β-Lactamase Inhibitor Combinations

Following initial MIC determination, the *in vitro* efficacies of the carbapenem/βLI combinations was assessed by adding fixed concentrations of the inhibitors to the experimental wells of a standard antimicrobial broth microdilution assay; thus, testing for Imipenem/βLI, Ertapenem/βLI, and Meropenem/βLI. The procedure followed in this assay adhered to CLSI guidelines; however, minor modifications to broth volumes were made in order to accommodate for the presence of the βLIs while keeping the concentrations of the carbapenems and bacterial suspensions in accordance with CLSI recommendations (Clinical and Laboratory Standards Institute [CLSI], 2012).

For isolates harboring OXA-type carbapenemases, Avibactam (MedChem Express, Monmouth Junction, NJ, United States) was used as the βLI at a fixed concentration of 4 μg/mL (Livermore et al., 2011; Aktas et al., 2012; Sader et al., 2015). Concerning the isolates that harbored *bla*_NDM-1_, ethylenediaminetetraacetic acid calcium disodium salt (calcium-EDTA) (Sigma^®^, St. Louis, MO, United States) was used as the βLI at a fixed concentration of 32 μg/mL (Aoki et al., 2010; Yoshizumi et al., 2013). As for the isolate that harbored *bla*_KPC-2_, Relebactam (MedChem Express, Monmouth Junction, NJ, United States) was used as the βLI at a fixed concentration of 4 μg/mL (Snydman et al., 2016).

In addition, each isolate was tested against its corresponding βLI at their aforementioned fixed concentrations without the addition of carbapenems in order to rule out any anti-bacterial activity exhibited by the inhibitors on the tested isolates.

The MICs of Imipenem (IPM), Ertapenem (ETP), and Meropenem (MEM) for all tested isolates were interpreted according to the CLSI M100 guideline (Clinical and Laboratory Standards Institute [CLSI], 2018a,b). MIC breakpoints for carbapenems in combinations with the βLIs used in this study are currently unavailable for *Enterobacteriaceae* and *A. baumannii*. As such, the MIC breakpoints for Ceftazidime/Avibactam (CAZ/AVI) were used to interpret the MIC results of the carbapenem/Avibactam combinations and the MIC breakpoints for IPM, ETP, and MEM alone were used to interpret the results of their combinations with Relebactam (REL) and Ca-EDTA.

As a quality control strain, *Escherichia coli* (ATCC^®^ 35218^TM^) was used according to CLSI recommendations for β-lactam/βLI combination testing (Clinical and Laboratory Standards Institute [CLSI], 2018c).

### Assessment of the *in vivo* Efficacy of Meropenem/β-Lactamase Inhibitor Combinations

The most efficacious *in vitro* treatment options that restored antimicrobial susceptibility of three isolates: *E. coli* IMP 57, *K. pneumoniae* IMP 216, and *Salmonella* spp. KPC, of which each harbored *bla*_OXA-48_, *bla*_NDM-1_, and *bla*_KPC-2_, respectively, were further investigated in animal experimentation models.

The animals involved in this study were purchased from the Animal Care Facility at the American University of Beirut. The protocols adopted in these experiments were reviewed and approved by the Institutional Animal Care and Use Committee (IACUC) at AUB under approval #17-08-432.

A total of 150 BALB/c male mice, 6–8 weeks old, weighing 20–40 g, were used in these sets of experiments. The mice were allowed to consume food and water *ad libitum* throughout the experimentation period and at the end of each set of experiments, all surviving mice were humanely euthanized.

#### Determination of the Median Lethal Dose in a BALB/c Murine Infection Model

The procedure followed in determining the LD_50_ of each of the isolates *E. coli* IMP 57, *K. pneumoniae* IMP 216, and *Salmonella* spp. KPC involved in the animal experimentations relied on an earlier protocol (Nowotny, 1979) with an extended monitoring period. Briefly, for each of the three tested bacterial isolates, 20 mice were divided into five groups of four mice. Each group of mice were intraperitoneally injected with increasing concentrations of the tested isolate, starting with 10^4^ CFU up to 10^8^ CFU. Following infection, the mice were daily monitored over a 1-week period for their survival, weight, physical appearance, and behavioral changes.

At the end of the monitoring period, the LD_50_ was calculated using the Spearman-Karber method (Nowotny, 1979).

#### Investigation of Survival Rates in a BALB/c Murine Infection Model

For each of the bacterial isolates *E. coli* IMP 57, *K. pneumoniae* IMP 216, and *Salmonella* spp. KPC, 30 mice were divided into five groups of six mice. The experimental setup was designed over a 7-day period (Table 3):

**Table 3:** Mice groups and injections used in survival experimentation.

Days		Group 1 (P.C.)	Group 2 (MEM)	Group 3 (MEM + βLI)	Group 4 (βLI)	Group 5 (N.C.)
Day 1	*t* = 0 hr	Bacterial injection	Bacterial injection	Bacterial injection	Bacterial injection	TSB
	*t* = 1 hr	–	Administer Meropenem	Administer Meropenem + β-lactamase inhibitor	Administer β-lactamase inhibitor	–
Day 2						
Day 3						
Day 4						
Day 5						
Day 6						
Day 7						


*The β-lactamase inhibitor (βLI) Avibactam was used against *E. coli* IMP 57 harboring *bla*_OXA-*48*_. The βLI calcium-EDTA was used against *K. pneumoniae* IMP 216 harboring *bla*_NDM-*1*_. The βLI Relebactam was used against *Salmonella* spp. KPC harboring *bla*_KPC-*2*_. TSB is tryptic soy broth; used as the injection solution for bacteria in Groups 1-4 and as a sterile blank in Group 5.*

1. Group 1 acted as a positive control and received a 1-time intraperitoneal bacterial dose of 3 × LD_50_ suspended in tryptic soy broth (TSB) on day 1 without receiving any subsequent treatment throughout the experiment.
2. Groups 2–4 received a 1-time intraperitoneal bacterial dose of 3 × LD_50_ suspended in TSB on day 1 followed by intraperitoneal treatment courses once, daily, starting 1-h post-infection on day 1 and continued days 2–7. Treatment for Group 2 was Meropenem only, for Group 3 was Meropenem/βLI, and for Group 4 was the corresponding βLI only.
3. Group 5 acted as a negative control and received a 1-time intraperitoneal injection of sterile TSB as a blank on day 1 without any subsequent injections throughout the experiment.

Meropenem (MEM) was used as the antimicrobial agent of choice in these sets of experiments as it was the most effective carbapenem *in vitro*. MEM was combined with Avibactam (AVI), calcium-EDTA (Ca-EDTA), and Relebactam (REL) against *E. coli* IMP 57, *K. pneumoniae* IMP 216 and *Salmonella* spp. KPC, respectively.

The required dose of MEM against the tested isolates was determined according to an earlier protocol (Rahal et al., 2011b) and was 1.6 mg/kg for *E. coli* IMP 57, 1.75 mg/kg for *Salmonella* spp. KPC, and 0.115 mg/kg for *K. pneumoniae* IMP 216.

As for the doses of the βLIs, AVI was administered at a 1:4 ratio with the antimicrobial agents (Endimiani et al., 2011; Levasseur et al., 2014), while Ca-EDTA and REL were each administered at an 8:1 ratio with the antimicrobial agents (Yoshizumi et al., 2013; Powles et al., 2018).

All mice were daily monitored for their survival, weight, physical appearance, and behavioral changes. Test subjects that expired prior to the end of the monitoring period had their blood cultured and API^®^ 20E (bioMérieux, Marcy l’Etoile, France) performed on the colonies retrieved in order to confirm that the cause of death was the administered agent (Salloum et al., 2015).

### Assessment of the Molecular Response to the Carbapenem/β-Lactamase Inhibitor Combinations

Reverse transcription real-time polymerase chain reaction (RT-qPCR) was used to quantitate the expression levels of *bla*_OXA-48_, *bla*_NDM-1_, and *bla*_KPC-2_ in the tested isolates. The relative normalized expressions of the target genes were calculated using the Livak 2^-ΔΔCT^ method (Schmittgen and Livak, 2008).

#### Under *in vitro* Conditions

Bacterial suspensions of *E. coli* IMP 57, *K. pneumoniae* IMP 216, and *Salmonella* spp. KPC were collected for RT-qPCR following their incubations with Meropenem alone as well as in combination with their corresponding βLIs at MICs. An untreated sample of each bacterial isolates was used as a positive control and the *rpoB* gene was used as a reference housekeeping gene (Salloum et al., 2015).

#### Under *in vivo* Conditions

For each of *E. coli* IMP 57, *K. pneumoniae* IMP 216, and *Salmonella* spp. KPC, 15 BALB/c male mice were divided into five groups of three mice and followed the same IACUC-approved infection and treatment protocols used in the survival studies above. However, mice from Groups 1–3 were then scarified via cardiac puncture under general anesthesia 4 h post-treatment, and their blood was collected for RT-qPCR. All blood samples were centrifuged at 1,500 × *g* for 30 min and the separated plasma was retrieved for bacterial RNA extraction (Rasheed, 2016).

For both *in vitro* and *in vivo* settings, the illustra^TM^ RNAspin Mini Kit (GE Healthcare UK Limited, Buckinghamshire, United Kingdom) was used to extract the RNA of each of the tested isolates, the iScript^TM^ cDNA Synthesis Kit (Bio-Rad, Hercules, CA, United States) was used to synthesize complementary DNA of the extracted RNA templates, and the iTaq^TM^ Universal SYBR^®^ Green Supermix (Bio-Rad, Hercules, CA, United States) was used for qPCR. All kits were utilized according to their manufacturers’ instructions. The real-time PCR primer sequences along with their amplicon sizes are available in Table 2.

### Statistical Analysis

The logrank (Mantel-Cox) test was utilized in the survival studies analysis to calculate the statistical significance while the unpaired Student’s *t*-test was used in the quantitative PCR analysis, in which *p*-values ≤ 0.05 were considered statistically significant.

## Results

### Detection of Carbapenem Resistance Genes

Following PCR amplification, it was observed that all collected isolates harbored at least 1 of the carbapenem resistance genes they were tested for. Consequently, *bla*_OXA-48_ was detected in five of the eight *Enterobacteriaceae* isolates (62.5%), *bla*_NDM-1_ was detected in 2 (25%), while *bla*_KPC-2_ was only detected in one *Enterobacteriaceae* isolate (12.5%). As for *bla*_OXA-23-like_ and *bla*_OXA-51-like_, they were amplified in all tested *A. baumannii* isolates (100%), while each of *bla*_OXA-58_ and *bla*_OXA-143-like_ were amplified in two of the six *A. baumannii* isolates (33.3%), and neither *bla*_OXA-24/40_ nor *bla*_NDM-1_ were detected in any of the tested *A. baumannii*. Concerning *bla*_NDM-1_ in *P. aeruginosa*, none of the tested isolates harbored the gene of resistance; thus, the *P. aeruginosa* isolates were not considered further in experimentation. A summary of the identified genes is available in Table 1.

### Efficacy of the Carbapenem/β-Lactamase Inhibitor Combinations *in vitro*

**FIGURE 1:**
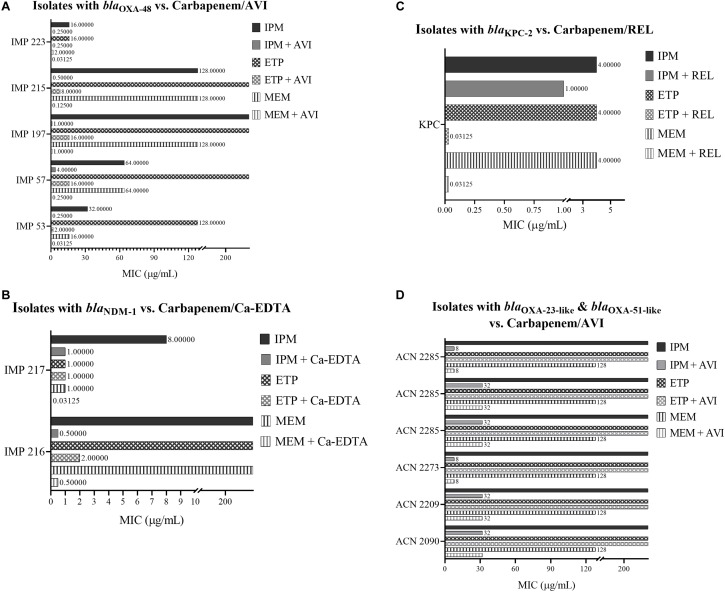
**(A)** MIC of carbapenems with and without Avibactam against *Enterobacteriaceae* isolates that harbor *bla*_OXA–48_. **(B)** MIC of carbapenems with and without Ca-EDTA against *Enterobacteriaceae* isolates that harbor *bla*_NDM–1_. **(C)** MIC of carbapenems with and without Relebactam against a *Salmonella* spp. isolate that harbors *bla*_KPC–2_. **(D)** MIC of carbapenems with and without Avibactam against *A. baumannii* isolates that mainly harbor *bla*_OXA–23–like_ and *bla*_OXA-51-like_.

Among the isolates that harbor *bla*_OXA-48_, IPM/AVI and MEM/AVI managed to restore carbapenem susceptibility to 100% of them while ETP/AVI restored carbapenem susceptibility to 60% of them. Similarly, among the isolates that harbor *bla*_NDM-1_, only testing with IPM/Ca-EDTA and MEM/Ca-EDTA resulted in 100% susceptibility, while for the isolate that harbored *bla*_KPC-2_, combining any of the carbapenems with REL restored carbapenem susceptibly. On the other hand, the combinations of any of the carbapenems with AVI were unsuccessful at restoring carbapenem susceptibility among the *A. baumannii* isolates that mainly harbored *bla*_OXA-23-like_ and *bla*_OXA-51-like_; however, they did manage to lower their MIC values by twofold in the case of ETP/AVI and at least eightfold in the cases of IPM/AVI and MEM/AVI. Minimal inhibitory concentration results of all isolates are available in Table 4 and Figure 1.

**Table 4:** MICs of Imipenem (IPM), Ertapenem (ETP), and Meropenem (MEM) with and without the β-lactamase inhibitors (βLI) against the tested isolates.

Gene of resistance	Isolate ID	MIC (μg/mL)					
		
		IPM	IPM + βLI	ETP	ETP + βLI	MEM	MEM + βLI
*bla*_OXA-48_	IMP 53	32	0.25	128	2	16	0.03125
	IMP 57	64	1	>256	16	64	0.25
	IMP 197	>256	1	>256	16	128	1
	IMP 215	128	0.5	>256	8	128	0.125
	IMP 223	16	0.25	16	0.25	2	0.03125
*bla*_NDM-1_	IMP 216	>256	0.5	>256	2	>256	0.5
	IMP 217	8	1	1	1	1	0.03125
*bla*_KPC-2_	KPC	4	1	4	0.03125	4	0.03125
*bla*_OXA-23-like_, *bla*_OXA-51-like_	ACN 2090	>256	32	>256	>256	128	32
	ACN 2209	>256	32	>256	>256	128	32
	ACN 2273	>256	8	>256	>256	128	8
	ACN 2285	>256	32	>256	>256	128	32
	ACN 2493	>256	32	>256	>256	128	32
	ACN 3630	>256	8	>256	>256	128	8


*The βLI for *bla*_*OXA-48*_, *bla*_*OXA-23-like*_, and *bla*_*OXA-51-like*_ is Avibactam. The βLI for *bla*_*NDM-1*_ is Ca-EDTA. The βLI for *bla*_*KPC-2*_ is Relebactam.*

Finally, none of the inhibitors used in this study were solely successful at inhibiting the growth of any isolate; thus, confirming that they do not exhibit antibacterial activities themselves.

### Efficacy of Meropenem/β-Lactamase Inhibitor Combinations *in vivo*

The median lethal dose of the tested isolates was determined as follows: 1.78 × 10^8^ CFU for *E. coli* IMP 57, 3.16 × 10^7^ CFU for *K. pneumoniae* IMP 216, and 3.16 × 10^8^ CFU for *Salmonella* spp. KPC. Recorded average mice weights are available in Supplementary Figure S1.

**FIGURE 2:**
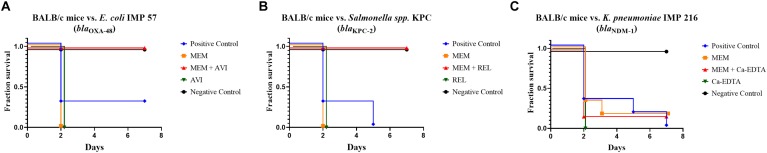
**(A)** Kaplan–Meier plot showing the survival rates of treated and untreated BALB/c mice infected with *E. coli* IMP 57. **(B)** Kaplan–Meier plot showing the survival rates of treated and untreated BALB/c mice infected with *Salmonella* spp. KPC. **(C)** Kaplan–Meier plot showing the survival rates of treated and untreated BALB/c mice infected with *K. pneumoniae* IMP 216.

Concerning the survival rate of the BALB/c mice upon infection with the tested isolates and treatment with Meropenem monotherapy in comparison to Meropenem/βLI combinations, the group receiving Meropenem/Avibactam against *E. coli* IMP 57 experienced a 100% survival rate (*p* < 0.0001) when compared to their positive control group (16.7% survival) as well as the group receiving Meropenem monotherapy (0% survival) and the group receiving Avibactam alone (0% survival) (Figure 2). Similarly, the group treated with Meropenem/Relebactam against *Salmonella* spp. KPC experienced a 100% survival rate (*p* < 0.0001) in comparison to their positive control group (0% survival) in addition to the group treated with Meropenem monotherapy (0% survival) and the group treated with Relebactam alone (0% survival) (Figure 2). However, the group receiving Meropenem/calcium-EDTA against *K. pneumoniae* IMP 216 experienced a 16.7% survival rate (*p* = 0.0009), identical to that of the group receiving Meropenem monotherapy, but higher than the positive control (0% survival) as well as the group receiving calcium-EDTA alone (0% survival) (Figure 2).

The average weights of the different mice groups against each of the tested bacterial isolates during the survival studies are available in Supplementary Figure S2.

### The Molecular Response to Meropenem/β-Lactamase Inhibitor Combinations

#### *bla*_OXA-48_

**FIGURE 3:**
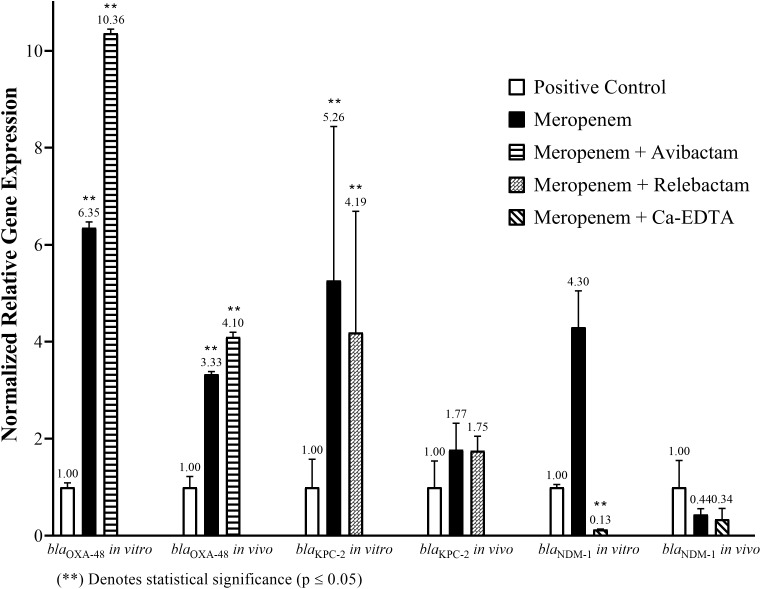
The *in vitro* and *in vivo* normalized relative gene expression levels of *bla*_OXA-48_ in *E. coli* IMP 57, *bla*_KPC-2_ in *Salmonella* spp. KPC, and *bla*_NDM-1_ in *K. pneumoniae* IMP 216 following the addition of Meropenem alone or in combination with Avibactam, Relebactam, and calcium-EDTA, respectively.

Quantifying the *in vitro* relative normalized expression levels of *bla*_OXA-48_ in *E. coli* IMP 57 following the addition of Meropenem only and Meropenem/Avibactam in comparison to the positive control indicated a sixfold increase (*p* = 0.0024) in *bla*_OXA-48_ expression when Meropenem was added, and a 10-fold increase (*p* = 0.00072) when Meropenem/Avibactam were added. Moreover, there was a statistically significant difference (*p* = 0.028) in expression levels when comparing Meropenem to Meropenem/Avibactam (Figure 3).

Concerning the *in vivo* relative normalized expression levels of *bla*_OXA-48_ in *E. coli* IMP 57, the treatment with Meropenem only and Meropenem/Avibactam in comparison to the positive control indicated a threefold increase (*p* = 0.0292) in *bla*_OXA-48_ expression when Meropenem was administered, and a fourfold increase (*p* = 0.0361) when Meropenem/Avibactam were administered (Figure 3).

#### *bla*_KPC-2_

Measuring the *in vitro* relative normalized expression levels of *bla*_KPC-2_ in *Salmonella* spp. KPC following the addition of Meropenem only and Meropenem/Relebactam, when compared to the positive control, indicated a fivefold increase (*p* = 0.008) in *bla*_KPC-2_ expression when Meropenem was added, and a fourfold increase (*p* = 0.05) when Meropenem/Relebactam were added (Figure 3).

With respect to the *in vivo* relative normalized expression levels of *bla*_KPC-2_ in *Salmonella* spp. KPC, the treatment with Meropenem only and Meropenem/Relebactam, when compared to the positive control, indicated a twofold increase in *bla*_KPC-2_ expression when either Meropenem alone (*p* = 0.43) or Meropenem/Relebactam (*p* = 0.29) were administered (Figure 3).

#### *bla*_NDM-1_

Quantifying the *in vitro* relative normalized expression levels of *bla*_NDM-1_ in *K. pneumoniae* IMP 216 following the addition of Meropenem only and Meropenem/calcium-EDTA when compared to the positive control indicated a fourfold increase (*p* = 0.134) in *bla*_NDM-1_ expression when Meropenem was added, but a significant eightfold decrease (*p* = 0.029) when Meropenem/calcium-EDTA were added. Moreover, there was a statistically significant difference (*p* = 0.021) in expression levels when comparing Meropenem to Meropenem/calcium-EDTA (Figure 3).

Quantifying the *in vivo* relative normalized expression levels of *bla*_NDM-1_ in *K. pneumoniae* IMP 216 following the treatment with Meropenem only and Meropenem/calcium-EDTA when compared to the positive control indicated 2.3-fold and 3-fold decreases in *bla*_NDM-1_ expression when Meropenem and Meropenem/Ca-EDTA were administered, respectively (Figure 3).

## Discussion

Antimicrobial resistance is a public health threat with major repercussions. Bacteria can rapidly develop resistance to new antimicrobial agents a few years after they become available for commercial use (Centers for Disease Control and Prevention [CDC], 2013). Carbapenem-resistant *Enterobacteriaceae* (CRE) and multidrug resistant *Acinetobacter baumannii* (MDR-*A. baumannii*) rank among the highest priority pathogens for research and drug discovery according to the (World Health Organization [WHO], 2017). Similarly, CREs are classified as an urgent health hazard, while MDR-*A. baumannii* is classified as a serious health hazard according to the (Centers for Disease Control and Prevention [CDC], 2013). Evidently, providing new and alternative solutions to treat carbapenem-resistant bacterial infections is a critical need. Although combination therapy has proven to be useful, its benefits over monotherapy remain debatable. Therefore, carbapenems/βLI combinations were chosen as potential alternative therapeutic solutions.

### Evaluating Avibactam

When assessing the *in vitro* capacity of carbapenem/Avibactam combinations against CREs and MDR-*A. baumannii*, the addition of the βLI to carbapenems successfully restored most of the tested isolates’ susceptibility to that class of antimicrobial agents.

The majority of the literature reports the combination of Ceftazidime/Avibactam against antimicrobial-resistant isolates; however, Aktas et al. (2012) have found that Imipenem/Avibactam managed to restore the susceptibility of 26 *Enterobacteriaceae* isolates with OXA-48. The findings reported in this study coincide with Aktas et al. (2012) regarding Imipenem/Avibactam; however, it was observed that the addition of Meropenem/Avibactam displayed considerably lower MIC values than the former combination (Table 4); thus, highlighting Meropenem/Avibactam as the more efficacious carbapenem/βLI combination against the tested *bla*_OXA-48_-positive *Enterobacteriaceae* isolates. On the other hand, Ertapenem was not as effective as Imipenem or Meropenem when combined with Avibactam against OXA-48 as it only managed to restore the susceptibility of three *Enterobacteriaceae* isolates. Finally, none of the carbapenem/Avibactam combinations used in this study managed to restore the susceptibility of any of the *A. baumannii* isolates that mainly harbored *bla*_OXA-23-like_ and *bla*_OXA-51-like_ (Table 1), which is consistent with the reported literature (Thaden et al., 2016).

Concerning the *in vivo* experiments, assessing the survival rate of murine infection models against *E. coli* IMP 57 that harbors *bla*_OXA-48_ and attempting to treat the animals with Meropenem/Avibactam has not been documented in the literature yet. As such, experimental design and dosage determinations were guided by earlier studies with similar target parameters (Rahal et al., 2011a; Levasseur et al., 2014; Salloum et al., 2015). The *in vitro* and *in vivo* results observed were compatible with minimal discrepancy. The group of mice that was infected with *E. coli* IMP 57 and treated with Meropenem/Avibactam showed a 100% survival rate, while that of the groups that received Meropenem or Avibactam monotherapy experienced a 0% survival rate (Figure 2). These findings prove the efficacy of Meropenem/Avibactam against OXA-48 among *Enterobacteriaceae*, and that the concentration of Meropenem to Avibactam at a 4:1 ratio is effective. Concerning the positive control group that was infected with *E. coli* IMP 57 without receiving any treatment, the survival of one mouse might have been due to a technical error during the intraperitoneal injection of the bacterial inoculum (Steward et al., 1968).

At the molecular level, both *in vitro* and *in vivo* results showed similar trends in relative *bla*_OXA-48_ expression, signifying the consistency of Meropenem/Avibactam activity despite the differences in environments. Meropenem appears to have induced the expression of *bla*_OXA-48_; however, its statistically significant overexpression upon the addition of Meropenem/Avibactam could be due to a synergistic relationship between Meropenem and Avibactam since it has been previously proven that Avibactam does not induce the production of β-lactamases by itself (Miossec et al., 2013), especially at concentrations below 32 μg/mL (Livermore et al., 2017); thus, had there not been synergism between them, the level of *bla*_OXA-48_ expression upon the addition of Meropenem/Avibactam would be similar to that following the addition of Meropenem alone. Additional data supporting the possibility of having synergism between Avibactam and Meropenem is their high affinity to the same penicillin-binding protein 2 (PBP2) in *E. coli* (Davies et al., 2008; Asli et al., 2016). It is worthy to note that regardless of the overexpressed carbapenemase, a low concentration of Avibactam was sufficient to inhibit the enzyme and permit the activity of Meropenem. It is possible that such a low concentration of Avibactam was sufficient due to the reversible inhibition property that it displays (Ehmann et al., 2012).

### Evaluating Relebactam

The *in vitro* testing of REL in combination with carbapenems has demonstrated its effectiveness against the tested *Salmonella* spp. KPC isolate as its susceptibility to IPM, ETP, and MEM was restored. These findings are in line with a previous study that reported IPM/REL as an effective combination against 78.5% Imipenem-non-susceptible non-*Proteeae Enterobacteriaceae* (Karlowsky et al., 2018). Furthermore, carbapenems/Relebactam showed targeted potency against KPC-2 and that also complements an earlier study that reported Imipenem/Relebactam restoring the susceptibility of 97% of KPC-producing *K. pneumoniae* isolates (Lapuebla et al., 2015). In addition, the safety and efficacy of Imipenem/Cilastatin/Relebactam versus Imipenem/Cilastatin alone have been tested in clinical trials among patients with complicated intra-abdominal infections (cIAI) (Lucasti et al., 2016) and complicated urinary tract infections (cUTI) (Sims et al., 2017). Both of those trials concluded that treating patients suffering from cIAI or cUTI using IPM/REL resulted in high rates of favorable microbiological and clinical responses at the end of treatment although IPM/REL was non-inferior to IPM alone. Despite the majority of the literature reporting Imipenem/Relebactam combinations, the results presented here provide Ertapenem and Meropenem as more efficacious alternatives as they displayed lower MICs, reaching 0.03125 μg/mL, upon their combination with Relebactam (Table 4).

With regards to the *in vivo* murine survival studies, the 100% survival rate of the mice treated with Meropenem/Relebactam against the *bla*_KPC-2_-positive bacterial isolate in comparison to the 0% survival rates of their control groups matched the *in vitro* results and mimicked those of Meropenem/Avibactam against *bla*_OXA-48_ (Figure 2), as this has also not yet been documented in the literature. These findings support the potency of the Meropenem/Relebactam combination against the tested *Salmonella* spp. KPC isolate *in vivo* as well as the efficacy of the 8:1 dosing ratio of Relebactam to Meropenem and the route of antimicrobial administration.

At the molecular level, Meropenem seemed to have induced the overexpression of *bla*_KPC-2_ *in vitro* regardless of the potentiating effect of Relebactam, as the latter is not a β-lactamase inducer by itself (Livermore et al., 2017); however, the insignificant decrease in gene expression levels due to the addition of Meropenem/Relebactam as compared to Meropenem alone could be due to the inhibitory effect that Relebactam exerted on KPC-2 without it having a compensatory mechanism that is similar to the one observed in *bla*_OXA-48_. On the other hand, the near-identical expression levels of *bla*_KPC-2_ *in vivo* upon the treatment with Meropenem monotherapy in comparison to Meropenem/Relebactam does not correlate with its *in vitro* gene expression observations despite displaying potency in the murine infection model. That discrepancy might be due to the change of environment between *in vitro* and *in vivo* settings, which could have altered the behavior of *bla*_KPC-2_ in response to treatment; however, that conclusion requires further investigation.

### Evaluating Calcium-EDTA

The *in vitro* assessment of Ca-EDTA in combination with carbapenems displayed high susceptibility rates amongst the tested isolates that harbor *bla*_NDM-1_ (Table 4). These results validate an earlier study that investigated the efficacy of Imipenem and Meropenem in combination with Ca-EDTA against NDM-1-positive *K. pneumoniae* and *E. coli* isolates (Yoshizumi et al., 2013); however, in contrast to that study, the Imipenem and Meropenem combinations with Ca-EDTA successfully lowered MIC values by a maximum of 512-fold, to reach 0.03125 μg/mL for Meropenem/Ca-EDTA, whereas those in Yoshizumi et al. (2013) were lowered by 256-fold at most, reaching 1 μg/mL. Finally, the combination of Ca-EDTA with Ertapenem was not as successful as with the other two carbapenems since it failed to render any of the tested isolates susceptible although it did lower the MIC of one isolate by at least 128-fold. These finding highlight Meropenem/Ca-EDTA as the more efficacious combination against the tested isolates.

Concerning the *in vivo* murine survival studies, Meropenem/Ca-EDTA did not demonstrate any added efficacy when compared to Meropenem monotherapy as the mice from both groups achieved identical survival rates of 16.7% whereas the positive control and Ca-EDTA monotherapy groups each resulted in 0% survival. A similar acute lethal septicemia experiment involving an NDM-1-positive *E. coli* isolate was performed by Yoshizumi et al. (2013); however, bacterial burden was assessed in that study and it was concluded that Imipenem/Ca-EDTA significantly reduced the bacterial burden in the blood and liver of neutropenic mice. In this study, the Meropenem/Ca-EDTA combination might have failed to demonstrate additional potency due to a potentially insufficient dose or an inappropriate intraperitoneal route of administration as Ca-EDTA might have chelated non-specific divalent cations found in the mice’s bodies.

With regards to the gene expression levels, the addition of Meropenem *in vitro* did not seem to significantly alter the expression of *bla*_NDM-1_; however, supplementing Ca-EDTA to Meropenem managed to significantly suppress the expression of the gene by approximately eightfold. These findings directly explain the observed decrease in MIC levels as Meropenem/Ca-EDTA successfully inhibited the carbapenemase and restored the isolate’s susceptibility. On the other hand, the use of Meropenem/Ca-EDTA as a treatment option in the murine infection model did not cause a significant difference in the expression levels of *bla*_NDM-1_ as compared to the positive control, but instead resulted in a gene expression level that is similar to the one due to Meropenem monotherapy. This latter observation supports the inefficacy of Meropenem/Ca-EDTA that was observed in the survival studies, as Ca-EDTA does not appear to have exerted its inhibitory effect on NDM-1 *in vivo*, leaving Meropenem to act on its own, which resulted in similar gene expression levels under both treatment conditions.

## Conclusion

Utilizing carbapenems, namely Meropenem, with the novel β-lactamase inhibitors Avibactam, Relebactam, and Ca-EDTA has proven to be capable of restoring carbapenem susceptibility among bacterial isolates that express the highly clinically relevant carbapenemases OXA-48, KPC-2, and NDM-1. Taken together, the *in vitro*, *in vivo*, and gene expression data encourage further investigating Meropenem/Avibactam and Meropenem/Relebactam as potential targeted therapeutic options against OXA-48 and KPC, respectively. However, a multi-faceted approach with a larger sample size and greater genetic diversity is required for both phenotypic and genotypic testing before proceeding into further preclinical and clinical development.

## Data Availability

All datasets generated for this study are included in the manuscript and/or the Supplementary Files.

## Ethics Statement

This study was carried out in accordance with the recommendations of the Institutional Animal Care and Use Committee at the American University of Beirut. The protocol was approved by the Institutional Animal Care and Use Committee at the American University of Beirut under approval #17-08-432.

## Author Contributions

BH designed and executed the experiments, analyzed the data, and wrote and edited the manuscript. SR designed the experiments, analyzed the data, and reviewed and edited the manuscript. AAF provided scientific feedback, analyzed the data, and reviewed and edited the manuscript. GA performed phenotypic bacteriological testing, supplied the bacterial isolates, provided scientific feedback, and reviewed and edited the manuscript. GM conceived the study, designed the experiments, analyzed the data, and edited the overall manuscript.

## Conflict of Interest Statement

The authors declare that the research was conducted in the absence of any commercial or financial relationships that could be construed as a potential conflict of interest.

## Supplementary Material

Figure S1 **(A)** Recorded average mice weights in their different groups during LD_50_ determination of *E. coli* IMP 57. **(B)** Recorded average mice weights in their different groups during LD_50_ determination of *K. pneumoniae* IMP 216. **(C)** Recorded average mice weights in their different groups during LD_50_ determination of *Salmonella* spp. KPC.

Figure S2 **(A)** Recorded average mice weights in their different groups during survival studies against *E. coli* IMP 57. **(B)** Recorded average mice weights in their different groups during survival studies against *K. pneumoniae* IMP 216. **(C)** Recorded average mice weights in their different groups during survival studies against *Salmonella* spp. KPC.
