# Supplementary figures and images for: Evaluating the Efficacies of Carbapenem/β-Lactamase Inhibitors Against Carbapenem-Resistant Gram-Negative Bacteria in vitro and in vivo
Source: Front Microbiol. 2019 Apr 30;10:933. doi: 10.3389/fmicb.2019.00933 (PMC6503214; doi:10.3389/fmicb.2019.00933)

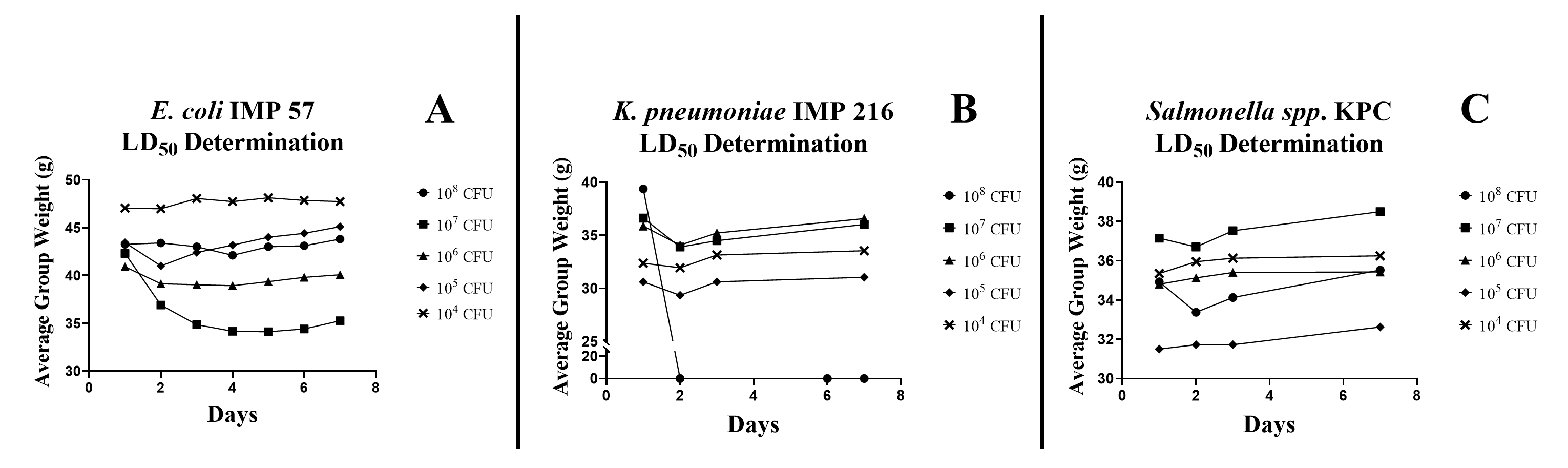

Supplement: Figure S1 — (A) Recorded average mice weights in their different groups during LD50 determination of E. coli IMP 57. (B) Recorded average mice weights in their different groups during LD50 determination of K. pneumoniae IMP 216. (C) Recorded average mice weights in their different groups during LD50 determination of Salmonella spp. KPC. [file Image_1.TIF]

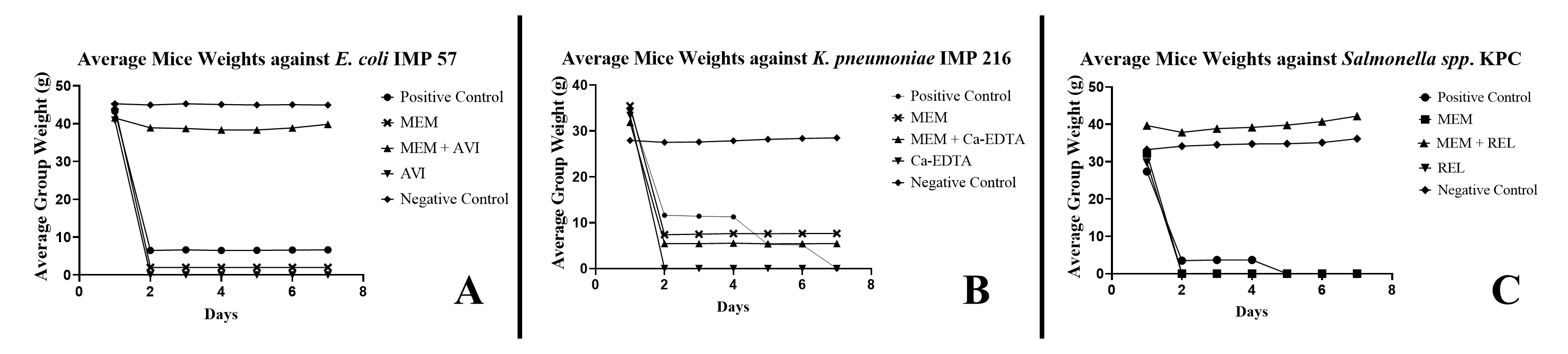

Supplement: Figure S2 — (A) Recorded average mice weights in their different groups during survival studies against E. coli IMP 57. (B) Recorded average mice weights in their different groups during survival studies against K. pneumoniae IMP 216. (C) Recorded average mice weights in their different groups during survival studies against Salmonella spp. KPC. [file Image_2.TIF]
